# Multicenter study on the effectiveness of the pre-epiglottic baton plate for airway obstruction and feeding problems in Robin sequence

**DOI:** 10.1186/s13023-017-0602-8

**Published:** 2017-03-09

**Authors:** Christian F. Poets, Christoph Maas, Wolfgang Buchenau, Joerg Arand, Anne Vierzig, Bert Braumann, Silvia Müller-Hagedorn

**Affiliations:** 1 Interdisciplinary Center for Craniofacial Malformations, Tuebingen University Hospital, Tuebingen, Germany; 2 Department of Neonatology, Tuebingen University Hospital, Calwerstrasse 7, Tuebingen, 72076 Germany; 3 Department of Pediatrics, Cologne University Hospital, Cologne, Germany; 4 Department of Orthodontics, Cologne University Hospital, Cologne, Germany; 5 Department of Orthodontics, Tuebingen University Hospital, Tuebingen, Germany

**Keywords:** Pierre-Robin sequence, Failure to thrive, Upper airway obstruction

## Abstract

**Background:**

Treatment of Robin sequence is often either invasive or of unproven effectiveness. The pre-epiglottic baton plate (PEBP) is a well-studied alternative, yet is not widely applied internationally. We report on a prospective 3-center cohort study investigating this treatment. Based on an agreed protocol, parents of infants with Robin sequence referred to participating centers were offered enrollment, which involved taking a maxillary cast followed by endoscopy to fit the plate and sleep studies to monitor its effectiveness. Recordings were centrally analyzed by sleep specialists blinded to timing and center. Primary outcome was the mixed-obstructive apnea index, defined as the number of such apneas/h of sleep; secondary outcomes included the desaturation index to <80% pulse oximeter saturation and weight gain.

**Results:**

Of 75 infants referred, 49 could be included; 1 center failed to perform appropriate sleep studies. Within a mean hospitalization of 3 weeks, the mixed-obstructive apnea index decreased (median; interquartile range) from 15.9 (6.3–31.5) to 2.3 (1.2–5.4); it decreased further to 0.7 (0.1–2.4) in the 32 infants who had a successful 3-month follow-up sleep study performed. The desaturation index normalized (from 0.38 (0–2.7) to 0.0 (0–0.1)). Mean standard deviation score for weight remained unchanged between admission and follow-up, while the proportion of tube-fed infants decreased from 74 to 14%.

**Conclusions:**

This prospective multi-center cohort study confirms retrospective audits on the effectiveness of PEBP treatment in improving upper airway obstruction and feeding problems, the main clinical problems of infants with Robin sequence. International collaboration is required to compare this with other treatment approaches.

**Trial registration:**

Number NCT02266043, Registered 30/09/2014; registered partially retrospectively.

## Background

Treatment of infants with the Robin sequence (RS), i.e. with retrognathia, glossoptosis, upper airway obstruction and optionally cleft palate, is dominated internationally by either rather invasive procedures, e.g. mandibular distraction osteogenesis, tongue-lip adhesion or mandibular traction, by bridging the narrow airway through a tracheostomy, use of a nasopharyngeal tube, or continuous positive airway pressure, or by recommending the prone position, which is of unclear effectiveness and fraught with an increased risk of sudden infant death syndrome [1]. In contrast, more than one third of RS infants born in Germany are treated with the pre-epiglottic baton plate (PEBP) according to a recent survey [2], which is a less invasive yet effective and comparatively well-studied alternative. This treatment, however, requires an interdisciplinary team with experience in upper airway endoscopy, pediatric sleep medicine, speech therapy and orthodontics, which is not easy to accomplish given that RS is a rare disease affecting only 1:8000–14000 infants [2]. As a result, published studies on PEBP treatment are yet from very few groups [3–5], calling into question the generalizability of the encouraging study results reported for this approach. Given this situation, colleagues from 3 centers, already offering treatment with the PEBP for several years, convened to perform an observational cohort study on the practicality and effectiveness of this treatment approach in RS infants referred to their centers.

## Methods

With funding from the German Ministry of Research and Education, orthodontists and neonatologists from the departments of pediatrics and orthodontics in Cologne (center 1), Tübingen (center 2) and Würzburg (center 3) met in November 2012 to harmonize their approach. In addition, and prior to study onset, 2 pediatricians from center 2 performed an initiation visit to the other centers to verify that the local infrastructure met the agreed standards. According to the study protocol, families of infants <1 year referred to participating centers were invited to take part in this observational study, i.e. all received the treatment considered standard of care in each center, only clinical and growth data as well as sleep study results were to be collected and analyzed centrally.

Infants were admitted and monitored in the neonatal intensive care unit, where they also underwent a multichannel baseline cardiorespiratory sleep study (polygraphy, PG). Next, infants had a maxillary imprint taken and a plaster cast was produced which was used for manufacturing a prototype of the PEBP. Using the cast as a model, palatal plates were made from hard acrylic (Forestacryl-Strong-S, Foerster, Pforzheim, Germany), covering both the palate (including the cleft) and the alveolar ridges. Once a prototype of the plate was ready, infants had an endoscopy to adjust the length and angle of the velar extension, which reached down to just above the epiglottis, with the angle of the baton being adjusted following endoscopy so that it pushed the base of the tongue sufficiently forward to open the pharynx. Plates were held in situ with the help of a fixative cream (Corega Super-Haftcreme; Procter & Gamble, Cincinnati, OH) and by extraoral wire bows secured on the infant’s face using adhesive tape (Steri-Strip and Cavilon-No Sting Barrier Film, Steri-Strip Compound Benzoin Tincture, 3 M Health Care, St. Paul, MN, USA). Indication for initiating PEBP treatment was a mixed-obstructive apnea index (MOAI) >3 [4]. The plate’s effectiveness in relieving upper airway obstruction was confirmed by further sleep studies, with the aim to achieve a MOAI <3. If this could not be achieved with the original PEBP, or if endoscopy showed a lateral or circular narrowing of the pharynx or the tongue compressing the velum against the posterior pharyngeal wall (i.e., a Sher type 2–4 obstruction [6]), either a plate with a steeper angle of the pharyngeal spur or one that had a perforated tube attached to the spur was used keep the pharyngeal space open. Infants received the final appliance for at least 48 h before the control sleep study used for this study was performed.

Treatment was supplemented by stimulation of the oral musculature, based on the Castillo-Morales® approach, and feeding training (initially via finger feeding, subsequently by bottle feeding with a nipple that allows controlling the ease of milk flow during sucking (Playtex Drop-Ins, Playtex Products, Edgewell, North Bergen, NY, USA). Infants were discharged from hospital once they had a MOAI <3 in a repeat sleep study and parents had demonstrated that they had learned how to insert, remove and clean the PEBP, which they were advised to do at least once daily. Follow-up visits including a sleep study had been planned approximately 3 months after hospital discharge, but performance of this sleep study could only be accomplished in center 2 (see Results). Further follow-up visits were at the discretion of participating centers, and were usually scheduled in 2–3 monthly intervals until cleft closure was scheduled at 10–12 months of age. Palatal plate treatment was discontinued once infants showed a normal tongue position and mandibular size (usually at 4–6 months of age). RS children are seen in the respective departments of orthodontics on an annual basis until reaching 16–18 years of age.

### Sleep studies

Sleep studies were performed using a computerized system (Embla N 7000, MedCare, Reykjavik, Iceland). The study montage comprised the following channels and sensors: chest and abdominal wall movements (respiratory inductive plethysmography, MedCare; Reykjavik, Iceland), nasal pressure and linearized nasal airflow (nasal prongs and built-in pressure transducer, MedCare), pulse oximeter saturation and pulse waveform (Radical, Masimo Inc., Irvine, USA), electrocardiogram (MedCare) and digital video via infrared camera (Panasonic; Tokyo, Japan). Recordings commenced in the evening and lasted for at least 8 h; all infants were studied in the supine position.

Recordings were manually analyzed, blinded to center and timing (before/during treatment/at follow-up), by pediatric sleep specialists from an external reference lab (Hospital Cologne-Porz) not otherwise involved in this study. Sleep study variables were classified using standard criteria [7, 8]. In brief, total sleep time (TST) was determined from the first 10-min epoch without movement, artifact or a distorted pulse waveform to the last such 10-min epoch; recordings comprising less than 3 h of TST were repeated during the following night. An apnea was scored if (i) the amplitude of the nasal airflow fell to <20% of the average amplitude of the two preceding breaths, (ii) no airflow was detected at the mouth, and (iii) the event comprised at least two breath cycles (i.e. approximately 4 s). An obstructive apnea was scored if (i) the above criteria for apnea were fulfilled and (ii) out-of-phase movements of the chest and abdomen were present. A central apnea was scored if (i) criteria for apnea were fulfilled and (ii) no chest and abdominal wall movements were present. Mixed apneas were defined as apneas with both central and obstructive components, each lasting at least two breath cycles. The MOAI was calculated as the sum of mixed and obstructive apneas per hour of TST; central apneas were not part of the MOAI.

Desaturation events were visually confirmed based on the pulse waveform signal to exclude spuriously low values. The number of desaturation events to ≤80% pulse oximeter saturation was counted and expressed as a desaturation index (DI_80_), defined as events per hour of TST.

### Ethics, consent and permissions

Institutional approval was given by each center’s ethics committee (reference number for Tübingen, the leading IRB: 160/2012BO1) and written informed parental consent obtained from caregivers of each infant enrolled in this study.

### Statistical analyses

Primary endpoint was the change in MOAI before vs. after treatment. Due to non-normally distributed data a sign-test was used. A *p*-value <0.05 was considered statistically significant concerning the primary endpoint. All other analyses are regarded descriptive. The following factors were analyzed for their possible influence on MOAI change between admission and discharge: gender (male vs. female), age at admission (≤28d vs. >28d) and failure-to-thrive (SDS for weight ≤1.28 vs. >1.28). All analyses were done with statistical software (SAS 9.2 for Windows; SAS; Heidelberg, Germany).

## Results

**Fig. 1:**
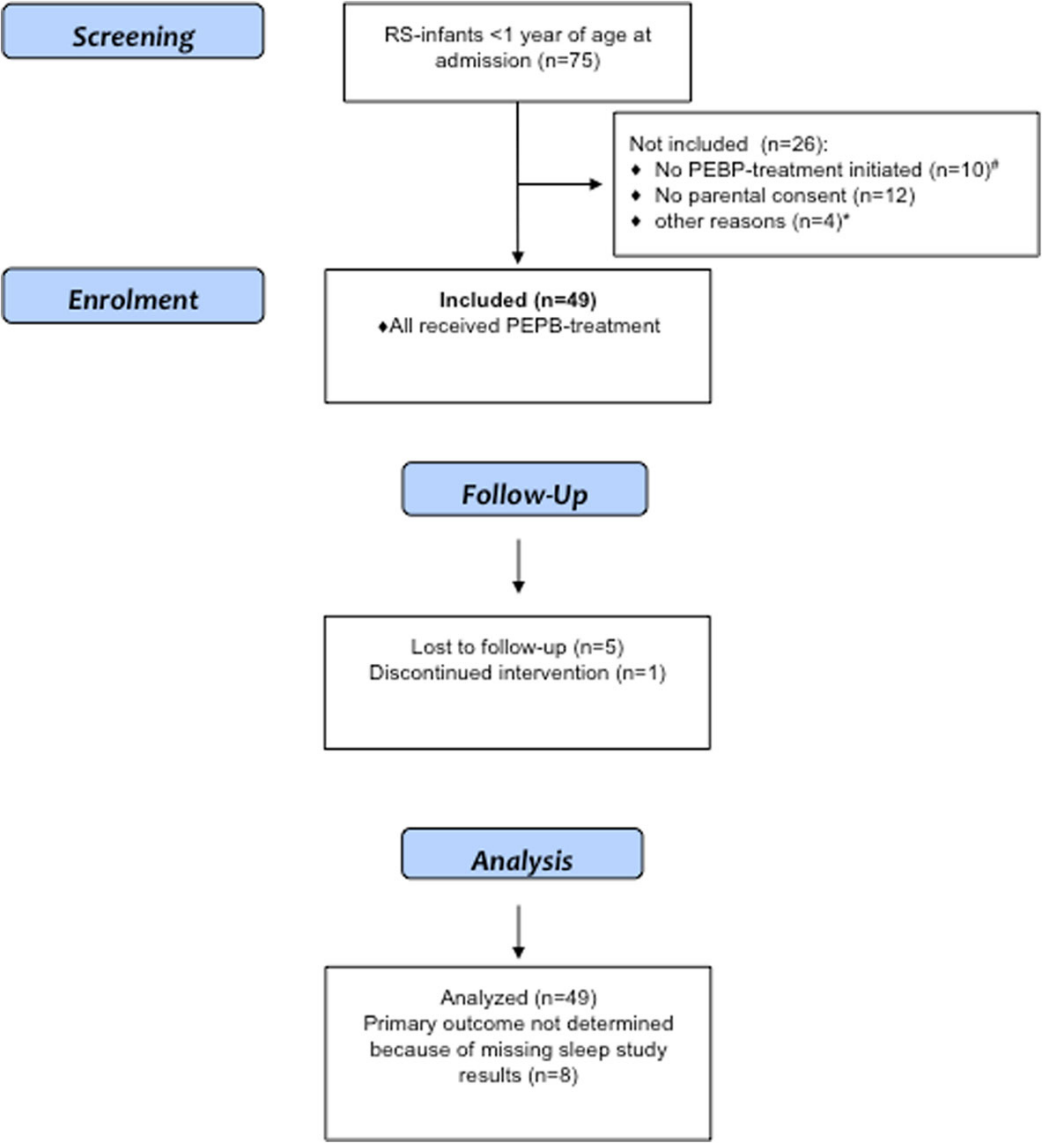
Patient Flow through this study. RS-Infants, infants with (Pierre-) Robin sequence; PEBP, pre-epiglottic baton plate. ^#^ In 7 infants with mild degree of upper airway obstruction a palatal plate without a spur was used, in 2 infants a CPAP-device or naso-pharyngeal tube was applied, 1 infant had early tracheostomy. ^*^ 1 study center (Würzburg) did not succeed in setting up and performing sleep studies, therefore the 4 patients referred to this center could not be included in the study

Between 11/02/2013 and 23/09/2015, 75 patients with RS < 1 year of age at admission were referred to participating centers, with 49 (24 boys) being included in this study: 8 (16%) from center 1 and 41 from center 2. Unfortunately, the third study center (Würzburg) did not succeed in setting up and performing sleep studies according to the agreed standards; thus, with no data on the primary outcome, no patient from this center could be included. Patient flow is depicted in Fig. 1; clinical variables are shown in Table 1. As there was no statistically significant difference in the primary outcome between center 1 and 2, results are shown for both remaining study centers together.

**Table 1:** Clinical data

Age at admission, mean (SD; interquartile range) (wk)	5.0 (5.4; 0.0–15.9)
Age at discharge, mean (SD; interquartile range) (wk)	8.0 (5.6; 1.1–6.1)
Age at follow-up, mean (SD; range) (wk)	18.6 (6.8; 7.0–36.1)
SDS for weight upon admission, mean (SD), *n* = 48	−0.84 (1.10)
SDS for weight at discharge, mean (SD), *n* = 49	−0.74 (0.87)
SDS for weight at follow-up, mean (SD), *n* = 41	−0.87 (0.96)
SDS for head circumference, upon admission; mean (SD), *n* = 49	−0.76 (1.27)
SDS for head circumference, at discharge; mean (SD), *n* = 48	−0.70 (1.17)
SDS for head circumference, at follow-up; mean (SD), *n* = 38	−0.57 (1.12)
Feeding tube upon admission, *N* (%)	36 (74%)
Feeding tube at discharge, *N* (%)	15 (31%)
Feeding tube at follow-up, *N* (%)	7 (14%)
Type of obstruction - Sher 1, *N*	40
- Sher 2, *N*	4
- Sher 3, *N*	3
- Not classified, *N*	2

Most infants (43; 88%) had isolated RS; syndromic RS was suspected in the remaining, with diagnoses including yet undefined syndrome (*N* = 3) and Franceschetti, Möbius and Emanuel syndrome (1 infant each). Forty-three (88%) infants had a cleft palate, and 47 (96%) were clinically assessed as having breathing difficulties upon admission. Prior to referral, infants had only received symptomatic treatment (e.g., prone positioning, nasopharyngeal tube). Endoscopically, the pharyngeal situation was classified as a Sher type 1 in the vast majority of infants (82%). Thus, the type of palatal plate used was the “classic” PEBP in 43 infants, and a plate with a perforated tube attached to the spur in 6 infants. In 1 infant, intermittently treated with the latter device, the MOAI improved so rapidly that she could be discharged on a plate without a spur. Most infants (32; 65%) had one plate fitted during their initial hospital stay, while 10 required 2, and five 3, different palatal plates. The remaining 2 infants had 4 and 6, respectively, plates fitted. No infant needed mechanical ventilation or required a tracheostomy.

Mean duration of hospital stay for orthodontic treatment was 3.0 (SD 2.0) weeks without significant differences between centers.

**Fig. 2:**
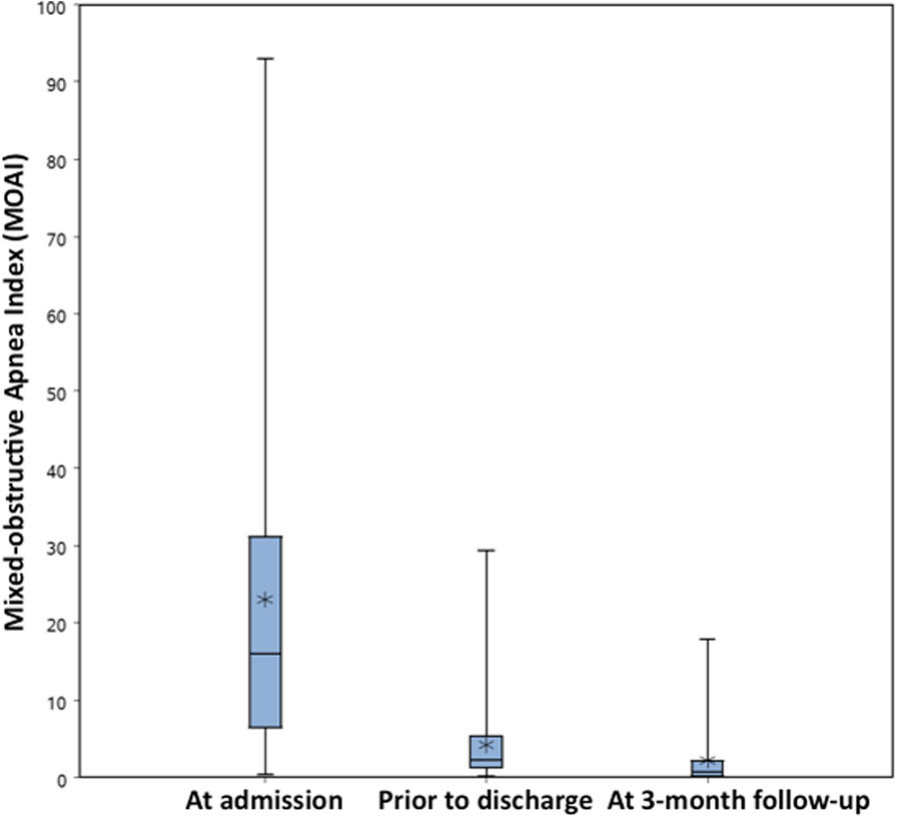
Boxplots showing results for the Mixed-Obstructive Apnea Index as the primary outcome of this study, studied upon admission, prior to hospital discharge, and at a 3-month follow-up visit

Upon admission, 46 of the 49 infants enrolled had an interpretable initial sleep study; data for the change in MOAI during hospitalization (the primary outcome) were available for 41 infants (Table 2). These showed a median decrease in the MOAI from 15.9 (interquartile range (IQR), 6.3–31.5) at admission to 2.3 (1.2–5.4) at discharge, *p* < 0.0001 (Fig. 2). 3-month follow-up data were available for 32 infants from Center 2 and showed a further decline in median MOAI to 0.7 (0.1–2.4). The DI_80_ also decreased (Table 2). Secondary analysis revealed no effect of gender (*p* = 0.1) or age at treatment onset (<28 days vs. > 28 days; *p* = 0.4) on the pre- vs. post-intervention change in MOAI. Furthermore, the MOAI did not differ between infants with vs. without failure-to-thrive at treatment onset (standard deviation score (SDS) for weight at admission < −1.28 vs. ≥ 1.28; *p* = 0.5).

**Table 2:** Sleep study results

Variable	Admission^a^	Discharge^b^	Follow-up^c^
MOAI, median (IQR)	15.9 (6.3–31.5) *n* = 46	2.3 (1.2–5.4) *n* = 44	0.7 (0.1–2.4) *n* = 32
DI80, median (IQR)	0.38 (0.0–2.68) *n* = 44	0.0 (0.0–0.13) *n* = 41	0.0 (0.0–0.08) *n* = 32

^a^ No data for 3 infants; ^b^ no data for 5 infants, *p* < 0,001 ^c^ no data for 17 infants

69% of infants were discharged without a feeding tube after initiation of PEBP-treatment with a remarkable difference between centers (38% at center 1 vs. 76% at center 2, *p* = 0.03). At 3-month follow-up, 2 of 41 infants treated at center 2, compared to 5 of 8 infants at center 1, still had a feeding tube in place. Mean SDS for weight between admission and 3-month follow-up remained nearly unchanged (−0.84 vs –0.87).

## Discussion

This prospective longitudinal cohort study confirms the effectiveness of the PEBP in improving upper airway obstruction and weight gain in infants admitted with (mostly isolated) RS. Similar to our previous retrospective and single-center cohort studies, we could now confirm that very similar improvements in upper airway obstruction, i.e. an 85% reduction in median MOAI within a few days of treatment, are possible in RS infants recruited prospectively and in 2 different centers. This was despite the fact that orthodontic staff at center 1 did not receive any formal training at the more experienced site, which confirms the ease of use of this minimally invasive orthodontic treatment. Furthermore, treatment success was ascertained in a setting with unbiased analysis of sleep studies. As expressed by weight gain along the original weight trajectory, PEBP treatment also helped to improve weight gain with mouth feeding: Although only 7 infants (14%) still had a feeding tube in place at the 3-month follow-up, mean SDS for weight remained nearly unchanged hence showing remarkably good weight growth velocity compared to other studies on infants with RS [9–11]. No infant suffered treatment failure or required switching to a surgical procedure to relieve residual airway obstruction, neither during the initial hospital stay nor at follow-up.

Nonetheless, our study also shows the difficulties involved in setting up and maintaining the multidisciplinary infrastructure required for an objective evaluation of any RS treatment, which should include repeated upper airway endoscopies and sleep studies [12]. Thus, despite considerable efforts, we failed to implement routine sleep studies in one center, and to perform 3-month follow-up studies at the other center. These failures may be characteristic of rare diseases, where it is difficult to maintain a complex infrastructure if only 2–3 patients/year are admitted. Here, center 2 was clearly at an advantage, as approximately 20% of all neonates with RS born in Germany are referred to this center [2]. Nonetheless, although not yet much used internationally, a recent epidemiologic survey showed that the PEBP approach studied here is already used in more than one third of RS infants admitted to hospitals in Germany [2].

There was a low proportion of syndromal RS patients in our cohort, and most had only a type-1 obstruction according to Sher [6]. Reasons for this are unclear, but several explanations may be considered: (i) most infants were referred at less than 6 weeks of age, bearing the potential for more subtle malformations to be overlooked; (ii) there was no formal standard at either center for the diagnostic work-up of these infants in the respective departments of genetics to identify syndromes potentially associated with RS; (iii) as published data on the effectiveness of PEBP treatment yet mostly involved infants with isolated RS, it is possible that there was some selection bias by referring hospitals, i.e. that infants with syndromal RS were less likely to be referred. In any case, our present data are only valid for infants with isolated RS.

Clearly, a controlled study design would have been preferable. This, however, was impossible in participating centers, as there was no longer equipoise among team members. However, we tried everything possible to minimize other potential sources of bias and thus consider our results valid against a background of studies performed in patients with rare conditions.

While the effectiveness of PEBP treatment in relieving upper airway obstruction did not differ between centers, there were differences in feeding and growth. Although not formally studied, we hypothesize that the considerable experience with high-calorie enteral feeding strategies [13] and orofacial stimulation therapy in center 2 [14] may have contributed to this result.

Contrary to our hypothesis, we could not confirm that infants starting treatment at an early age had a more pronounced improvement in MOAI than those starting beyond the neonatal period. Nonetheless, we consider it important to know whether PEBP treatment does induce mandibular catch-up growth, for which the largest potential would be expected in early infancy, and will address this in future studies.

## Conclusion

In this first prospective and multicenter study on the effectiveness of PEBP treatment in improving UAO and weight gain in infants referred for (largely isolated) RS, we could confirm retrospective and single-center studies that it does. However, our data allow no comparison to other treatment approaches such as mandibular distraction osteogenesis or the nasopharyngeal airway, so no recommendation concerning the best approach is possible. Given the heterogeneity in treatment approaches to RS seen world-wide, with strong beliefs held by each team in their respective approach, we suggest to set up an international collaboration among national reference centers to define a common protocol for comparing the effectiveness of each approach in their patient population, e.g. using sleep studies and weight charts.

## Abbreviations

DI: Desaturation index
MOAI: Mixed-obstructive apnea index
PEBP: Pre-epiglottic baton plate
RS: Robin sequence
SDS: Standard deviation score
TST: Total sleep time
UAO: Upper airway obstruction
